# Metformin Inhibits Cyst Formation in a Zebrafish Model of Polycystin-2 Deficiency

**DOI:** 10.1038/s41598-017-07300-x

**Published:** 2017-08-02

**Authors:** Ming-Yang Chang, Tsu-Lin Ma, Cheng-Chieh Hung, Ya-Chung Tian, Yung-Chang Chen, Chih-Wei Yang, Yi-Chuan Cheng

**Affiliations:** 1 Kidney Research Center and Department of Nephrology, Chang Gung Memorial Hospital, Chang Gung University College of Medicine, Taoyuan, Taiwan; 2 Graduate Institute of Biomedical Sciences, College of Medicine, Chang-Gung University, Taoyuan, Taiwan

## Abstract

Autosomal dominant polycystic kidney disease (ADPKD) is a common kidney disease caused by mutations in *PKD1* or *PKD2*. Metformin reduces cyst growth in mouse models of *PKD1*. However, metformin has not been studied in animal models of *PKD2*, and the cellular mechanism underlying its effectiveness is not entirely clear. This study investigated the effects of metformin on cyst formation in a zebrafish model of polycystin-2 deficiency resulting from morpholino knockdown of *pkd2*. We added metformin (2.5 to 20 mM) to the embryo media between 4 and 48 hours post fertilisation and observed pronephric cyst formation by using the *wt1b* promoter-driven GFP signal in *Tg*(*wt1b:GFP*) *pkd2* morphants. Metformin inhibited pronephric cyst formation by 42–61% compared with the untreated controls. Metformin also reduced the number of proliferating cells in the pronephric ducts, the degree of dorsal body curvature, and the infiltration of leukocytes surrounding the pronephros. Moreover, metformin treatment increased the phosphorylation of adenosine monophosphate-activated protein kinase (AMPK) and enhanced autophagy in the pronephros. Our data suggest that metformin reduces cyst formation through activation of the AMPK pathway and modulation of defective cellular events such as proliferation and autophagy. These results also imply that metformin could have therapeutic potential for ADPKD treatment.

## Introduction

Autosomal dominant polycystic kidney disease (ADPKD) is a common genetic kidney disease resulting from mutations of *PKD1* or *PKD2* ^1^. Affected patients may gradually lose their renal function because of progressive cyst formation in the kidneys; ADPKD accounts for 5% to 10% of cases of end-stage kidney disease (ESKD)^2^. Extrarenal presentations, such as liver cysts, cerebral aneurysms, and colon diverticulosis, can also cause serious complications. Patients with *PKD2* mutations have a milder disease course than do those with *PKD1* mutations. The ages at which patients with *PKD1* and *PKD2* mutations develop ESKD differ by almost 20 years^3^.

Polycystin-1 (encoded by *PKD1*) is a 4302-amino-acid membrane glycoprotein that is responsible for cell–cell or cell–matrix interactions critical for maintaining the renal tubular morphology^4^. Polycystin-2 (encoded by *PKD2*) is a 968-amino-acid cation ion channel involved in the regulation of intracellular calcium homeostasis^5, 6^. Polycystin-1 and polycystin-2 can act synergistically or independently at the cell membrane, endoplasmic reticulum, and cilia, regulating cell proliferation, fluid secretion, extracellular matrix mechanics, and ciliary functions^7^.

The mechanism of cyst formation in ADPKD remains incompletely understood. Previous studies have shown that cyclic adenosine monophosphate (cAMP) and mammalian target of rapamycin (mTOR) are the major pathways involved in cell proliferation and fluid secretion in ADPKD^8^. Recent studies have suggested that enhanced aerobic glycolysis and defective autophagy in ADPKD cells may contribute to its hyperproliferative phenotype^9^. Furthermore, experimental evidence suggests that inflammation and macrophage infiltration have an additional role in promoting cystogenesis^10^. These novel discoveries of disease mechanisms have led to the investigation of many targeted therapies^11–13^. However, no treatment for ADPKD approved by the US Federal Drug Administration is currently available. A possible strategy to accelerate the discovery of new treatment is identifying new uses of existing drugs and repurposing them accordingly.

Metformin, an approved biguanide derivative, has been used for treating type 2 diabetes mellitus for decades^14, 15^. Previous studies have shown that metformin inhibits gluconeogenesis through the activation of liver kinase B1 and adenosine monophosphate-activated protein kinase (AMPK)^16, 17^. In recent years, the therapeutic potential of metformin in cancer and polycystic ovary disease has drawn increasing attention^18, 19^. For example, data from retrospective studies indicate that patients treated with metformin have a lower risk of prostate cancer and other urologic malignancies^20^, suggesting that metformin could have clinical applications in addition to glycemic control in type 2 diabetes mellitus^21^.

MacCarty *et al*. hypothesised that metformin can reduce cyst formation on the basis of its abilities to activate AMPK and suppress cystic fibrosis transmembrane conductance regulator (CFTR) and mTOR^22^. Accordingly, a previous study demonstrated that metformin inhibited cyst growth in Madin–Darby canine kidney (MDCK) cells and *Pkd1* conditional knockout mice^23^. However, the exact *in vivo* mechanisms underlying the effect of metformin on cystogenesis are not entirely understood. Furthermore, recent studies have suggested that polycystin-1-deficent and polycystin-2-deficent cells might be different in the degree of AMPK inhibition^24^. Hence, whether metformin can inhibit cyst growth in the setting of polycystin-2 deficiency and the possible mechanisms of its action remain to be determined.

In this study, we investigated the effects of metformin on the initiation of pronephric cysts using a zebrafish *pkd2* model. We observed that metformin reduces pronephric cyst formation through the activation of AMPK and restores autophagy activity. These findings indicate that metformin could play a role in the treatment of early-stage ADPKD.

## Results

### Metformin prevents cyst initiation in *pkd2* morphants

**Figure 1:**
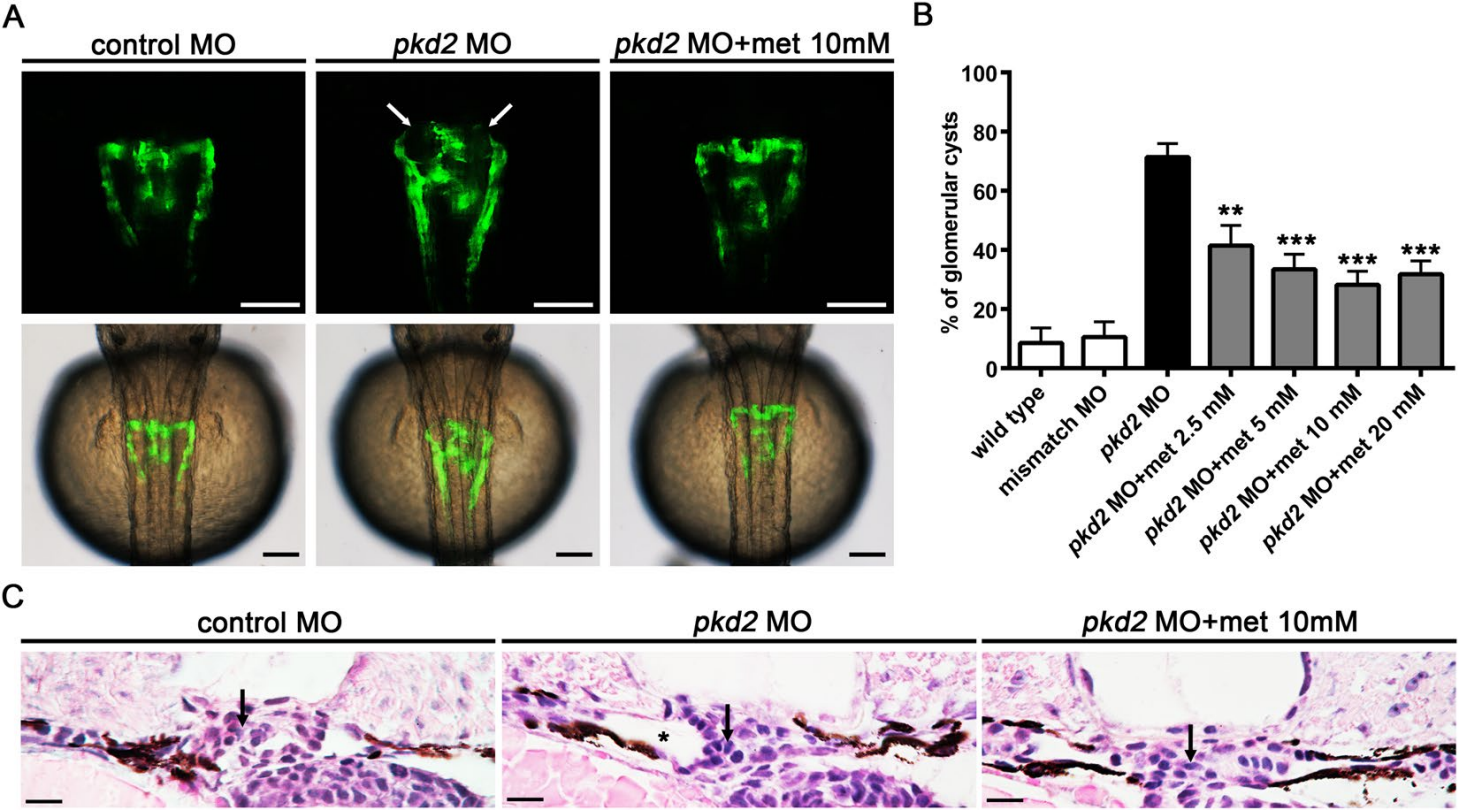
Metformin inhibits pronephric cyst formation in *pkd2* morphants. *Tg*(*wt1b:GFP*) *pkd2* morphants were immersed in embryo media (E3) supplemented with 2.5, 5, 10, or 20 mM metformin until 48 hpf. (**A**) Representative images of the pronephros in the control embryos, *pkd2* morphants, and metformin-treated *pkd2* morphants are shown. Note the pronephric cysts (arrows) in the *Tg*(*wt1b:GFP*) *pkd2* morphant. Photographs from *in vivo* observation through fluorescence microscopy (dorsal view, anterior to the top) were recorded at 48 hpf. The lower panels show overlays of transmission (grey) and fluorescence (green) images of the upper panels. Scale bar, 100 µm. (**B**) Comparative frequencies of the cystic phenotype in the control embryos and *pkd2* morphants treated with different concentrations of metformin as indicated (n = 45–99 per group). Data represent five independent experiments. **P < 0.01, ***P < 0.001 compared with *pkd2* knockdown. (**C**) Transverse histological sections of the embryos at 48 hpf, illustrating the pronephric cyst (indicated by the mark *) in a *pkd2* morphant and the rescue effect of metformin treatment. Arrows indicate the position of glomeruli. H&E stain, scale bar, 10 µm.

In this study, we used a zebrafish model of PKD2 for evaluating the effects of metformin on cyst initiation^25–29^. A translation-blocking morpholino oligos (MO) against *pkd2* was injected in a transgenic line, in which green fluorescent protein (GFP) expression was driven by the pronephros-specific *wt1*b promoter. We treated the *Tg*(*wt1b:GFP*) *pkd2* morphants with different concentrations of metformin in the E3 buffer between 4 and 48 hours post fertilisation (hpf). As illustrated in Fig. 1A, metformin treatment resulted in a significant reduction (42% to 61%) of cyst formation in the *Tg*(*wt1b:GFP*) *pkd2* morphants compared with untreated controls at 48 hpf (*P* < 0.01). The frequency of glomerular cyst formation decreased dose-dependently from 71.4% ± 4.6% in the untreated *pkd2* morphants to 41.5% ± 6.8% with 2.5 mM metformin and 33.4% ± 5.1%, 28.2% ± 4.6%, and 31.8% ± 4.5% with 5 mM, 10 mM, and 20 mM metformin, respectively (Fig. 1B). In transverse histological sections, we confirmed the rescue effect of metformin treatment on the pronephric cysts in the *pkd2* morphants (Fig. 1C). These results indicate that metformin could inhibit the early stage of cyst formation in the zebrafish model of PKD2.

### Metformin reduces dorsal body curvature and cloaca malformation in *pkd2* morphants

**Figure 2:**
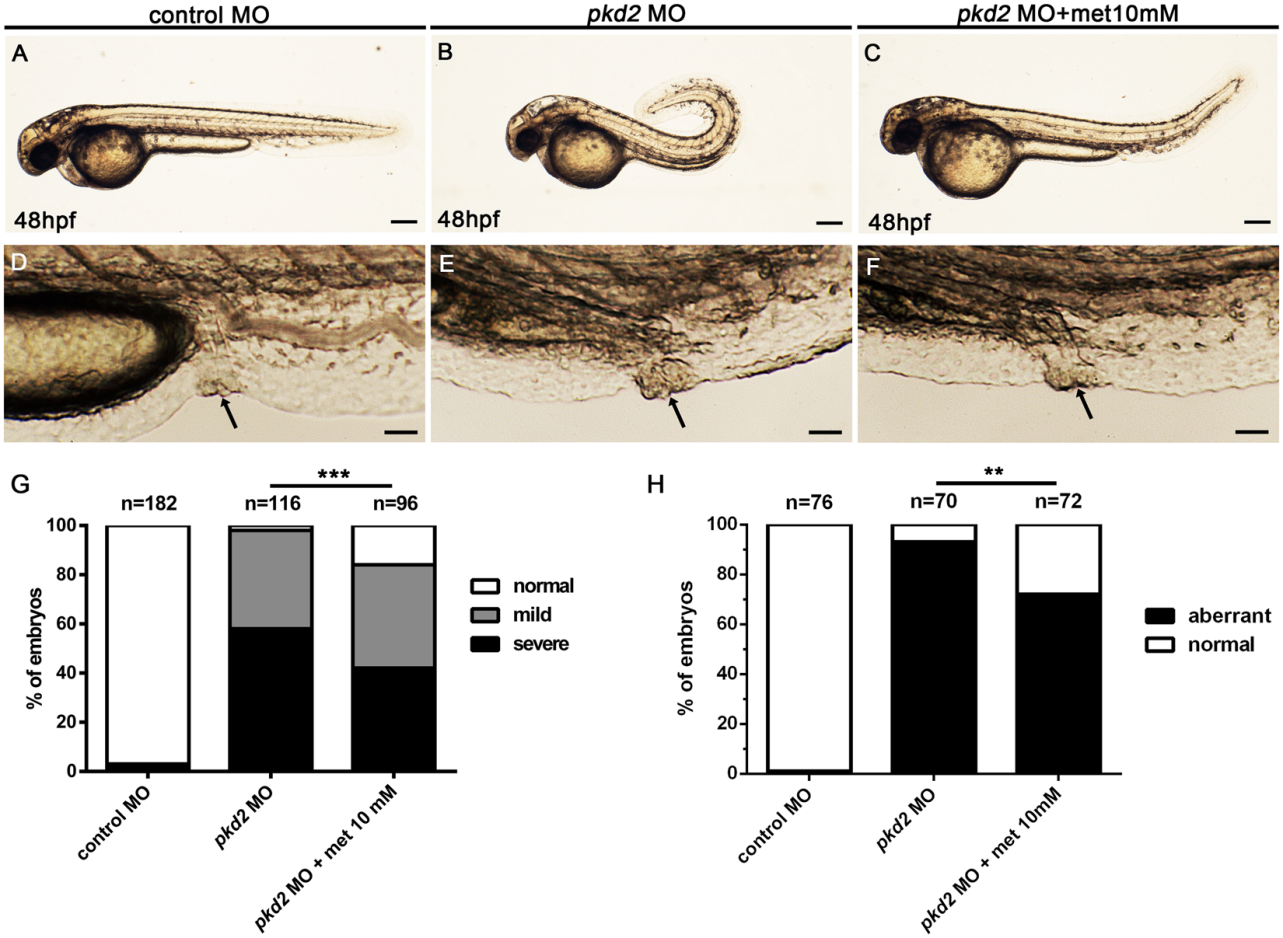
Metformin ameliorates dorsal axis curvature and cloaca malformation in *pkd2* morphants. Zebrafish embryos were treated with metformin (10 mM) in supplemented E3 media. (**A**–**C**) Representative images showing the common body axis of a control embryo, the severe dorsal curvature of a *pkd2* morphant, and the mild dorsal curvature of a metformin-treated *pkd2* morphant. Scale bar, 200 µm. (**D**–**F**) Representative images showing the cloaca of a control embryo, the cloaca malformation of a *pkd2* morphant, and the normal cloaca of a metformin-treated *pkd2* morphant. Scale bar, 50 µm. (**G**) Comparative frequencies of the dorsal curvature phenotype in each group (n = 182, 116, and 96 per group). Data represent four independent experiments. ***P < 0.001 compared with *pkd2* morphants. (**H**) Comparative frequencies of the aberrant cloaca phenotype in each group (n = 76, 70, and 72 per group). Data represent three independent experiments. **P < 0.01 compared with *pkd*2 morphants.

We examined the effect of metformin on the curvature phenotype of *pkd2* morphants (Fig. 2A–C). Dorsal axis curvature results from an overproduction of type II collagen in the notochord sheath and has been used as a surrogate readout for cystogenesis in *pkd2* morphants^26^. As illustrated in Fig. 2G, metformin partially reduced the frequencies of the overall curvature and severe dorsal curvature (>90°) phenotypes by 14% (114/116 vs. 81/96, *P* < 0.001) and 15% (67/116 vs. 41/96, *P* < 0.05), respectively, compared with untreated controls. These data indicate that metformin also ameliorated the defects in extracellular matrix formation involved in the pathogenesis of ADPKD. For comparison, we treated *pkd1a*/*b* morphants with 10 mM metformin and the curvature phenotype was also partially suppressed, similar to that observed in *pkd2* morphants (Supplementary Fig. S1). However, the small percentages of pronephric cysts in the *pkd1a*/*b* morphants preclude us from further studies using this model^30^.

The cloaca malformation has been shown to correlate the formation of pronephric cysts in several zebrafish models of ciliopathies^6, 31–33^. In agreement with these studies, we found that the frequency of aberrant cloaca increased in *pkd2* morphants and metformin significantly improved the phenotype by 21% (65/70 vs. 52/72, *P* < 0.01) (Fig. 2D–F,H). These data indicate that metformin consistently suppressed the different phenotypes of *pkd2* morphants. However, whether the improvement in cloacal morphology can lead to increased urine flow in metformin-treated *pkd2* morphants requires further study of kidney function using rhodamine-dextran filtration assays^33–36^.

### Metformin reduces tubular cell proliferation

**Figure 3:**
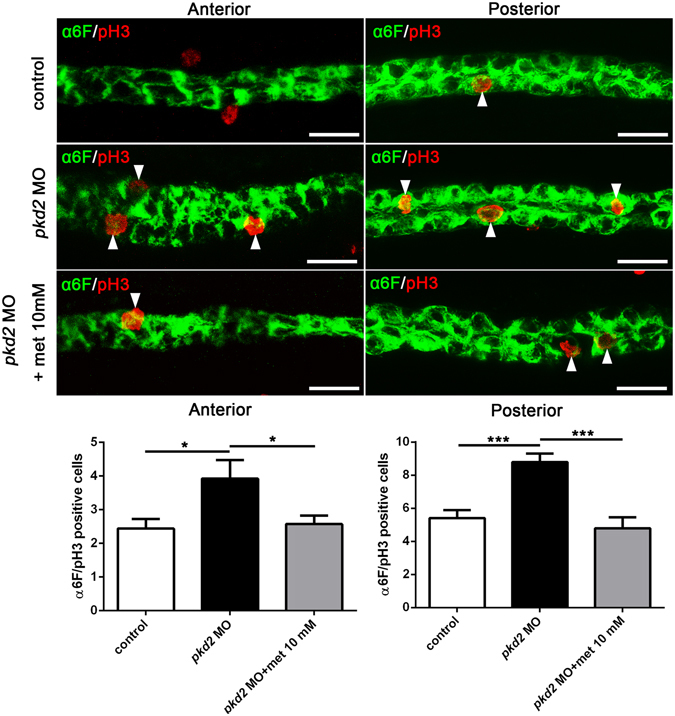
Metformin suppresses pronephric epithelial proliferation in *pkd2* morphants. (**A**) Representative confocal immunofluorescence images showing proliferative cells (arrows) in *pkd2* morphants with and without metformin (10 mM) treatment. Embryos were stained using anti-PH3 (red) to mark proliferating cells and anti-α6 F (green) to label the pronephric ducts at 48 hpf. (**B**) Counts of anti-PH3- and anti-α6F-positive cells in the anterior and posterior pronephric ducts (n = 14–17 per group). *P < 0.05, ***P < 0.001. Data represent two independent experiments. Scale bar, 20 µm.

We next sought to determine whether metformin affects cell proliferation in the pronephric kidney. We performed double immunostaining using antibodies against phosphohistone H3 (PH3) and the Na/K-ATPase α-1 subunit (α6 F) to mark proliferating cells in the pronephric ducts (Fig. 3A). Significantly fewer proliferating cells were observed in the pronephric ducts of the *pkd2* morphants treated with metformin than in those of the untreated *pkd2* morphants (Fig. 3B). These data suggest that metformin reduced pronephric cyst formation in the *pkd2* morphants through inhibition of epithelial cell proliferation.

### Metformin reduces leukocyte accumulation

**Figure 4:**
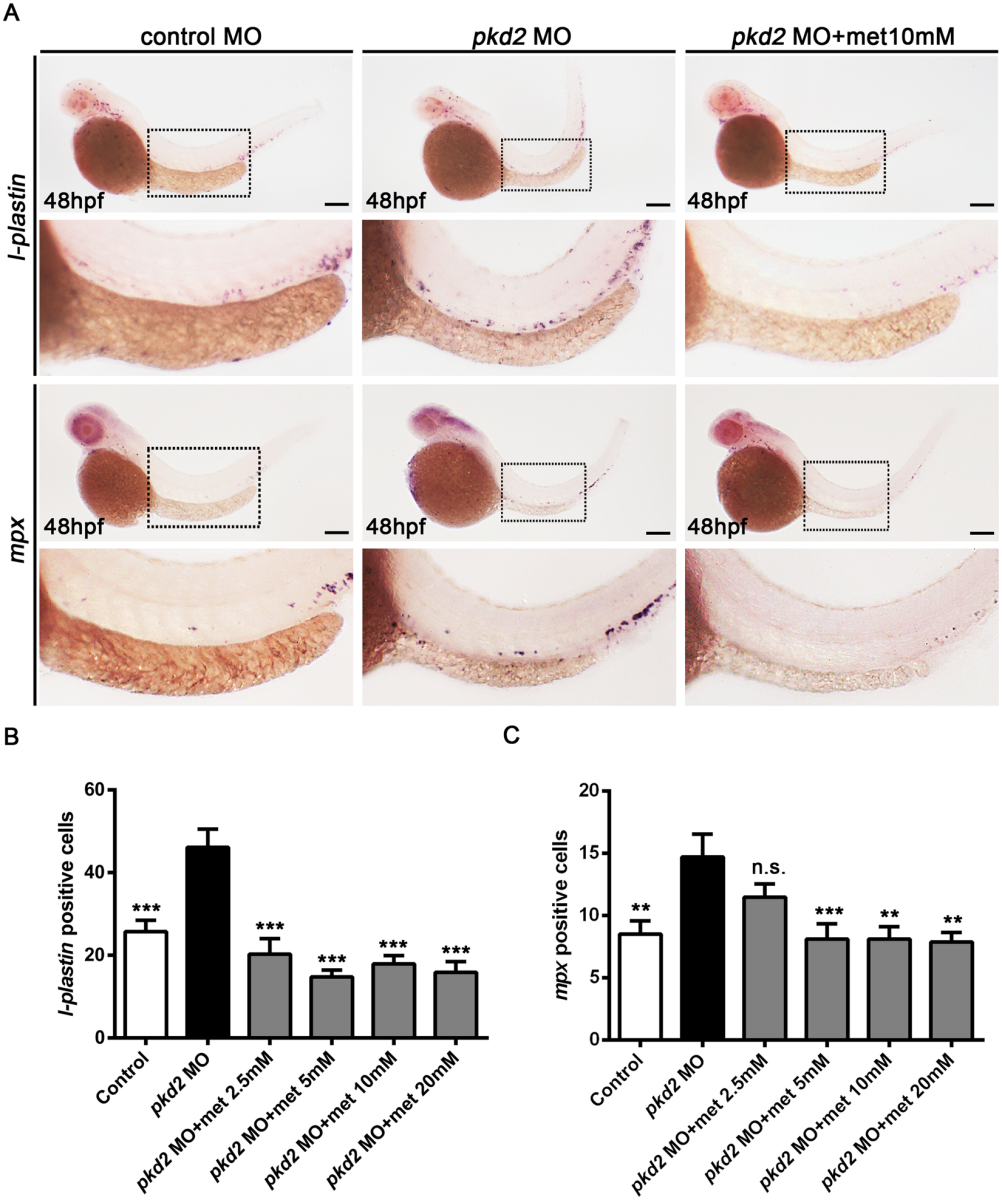
Metformin reduces leukocyte infiltration in *pkd2* morphants. (**A**) Representative images of *l-plastin*-positive and *mpx-*positive cells surrounding the pronephric ducts in a control MO-injected embryo, a *pkd2* morphant, and a *pkd2* morphant treated with metformin (10 mM). Leukocytes were labelled using *in situ* hybridisation for *l-plastin* and *mpx* at 48 hpf. The lower panel shows an enlarged view of the dashed box indicated in the upper image. Scale bar, 200 µm. (**B**) Counts of the *l-plastin*-positive cells (within the region of yolk extension as indicated by the dashed box) in the controls and metformin-treated *pkd2* morphants (2.5 mM to 20 mM). ***P < 0.001 compared with *pkd2* morphants. Data represent two independent experiments, n = 7–11 per group. (**C**) Counts of the *mpx*-positive cells. **P < 0.01, ***P < 0.001 compared with pkd2 morphants. n.s. = not significant. Data represent two independent experiments, n = 16–20 per group.

Previous studies have revealed that inflammation contributes to cell proliferation and cyst formation in ADPKD^10^. To explore the mechanisms by which metformin inhibits cyst growth, we analysed the effect of metformin on leukocyte infiltration in the *pkd2* morphants. Whole-mount *in situ* hybridisation revealed significantly increased *l-plastin* and *mpx* expression in the trunk area surrounding the pronephric ducts in the *pkd2* morphants compared with mismatched MO controls (Fig. 4A). The pan-leukocyte marker *l-plastin* is an actin-binding protein preferentially expressed in monocytes/macrophages and *mpx* is a neutrophil marker^37^. Metformin treatment caused a significant reduction of leukocyte infiltration in the pronephric area in the *pkd2* morphants (Fig. 4B and C). These data indicate that metformin could have an additional anti-inflammatory role that contributes to its therapeutic effects on polycystic kidney disease.

### Activation of AMPK by metformin

**Figure 5:**
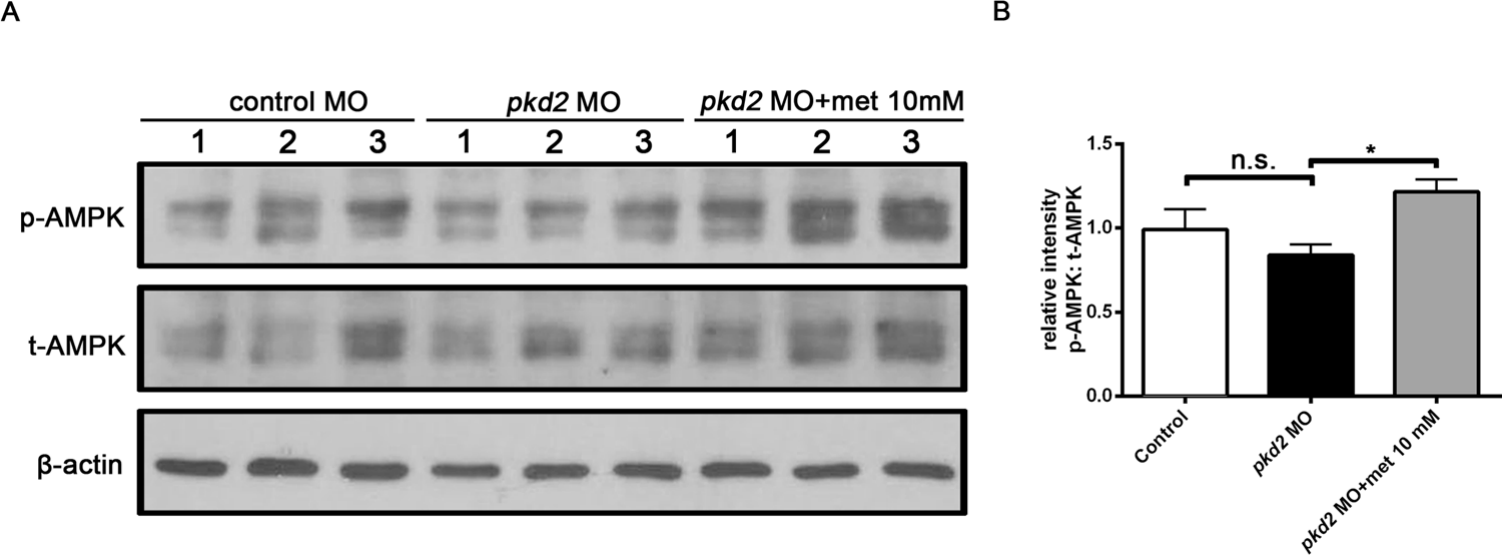
Metformin increases AMPK levels in *pkd2* morphants. (**A**) Representative Western blots for phospho-Thr^172^ AMPK, total AMPK, and β-actin in total embryo lysates from mismatch MO-injected controls and *pkd2* morphants with and without metformin (10 mM) treatment at 48 hpf. (**B**) Quantification of p-AMPK relative to total AMPK through densitometric analysis of Western blots; n = 9 from five independent experiments. *P < 0.05, n.s. = not significant. Cropped blot images are shown, and all the blots were run under the same experimental conditions. Uncropped blots are shown in Supplementary Fig. S2.

A previous study revealed that *Pkd1* ^−/−^ mouse embryonic fibroblasts exhibit a lower level of AMPK phosphorylation than do wild-type cells^9^. Metformin has been shown to stimulate AMPK in cell and mouse models of ADPKD^23, 24^. Therefore, we evaluated the effect of metformin on the activation of the AMPK signalling pathway. Western blot analysis using an antibody against p-AMPKα at Thr172 indicated that the p-AMPK/AMPK ratio was significantly higher in the metformin-treated *pkd2* morphants than in the untreated controls (Fig. 5A and B, Supplementary Fig. S2). These data are consistent with the hypothesis that AMPK activation inhibits polycystic kidney disease. However, we did not observe a lower baseline expression level of p-AMPK in the *pkd2* morphants than in the wild-type controls; this effect could be partly due to the presence of residual maternal pAMPK in the zebrafish embryos^38^.

### Metformin cannot prevent cyst formation in *tsc1a* morphants

**Figure 6:**
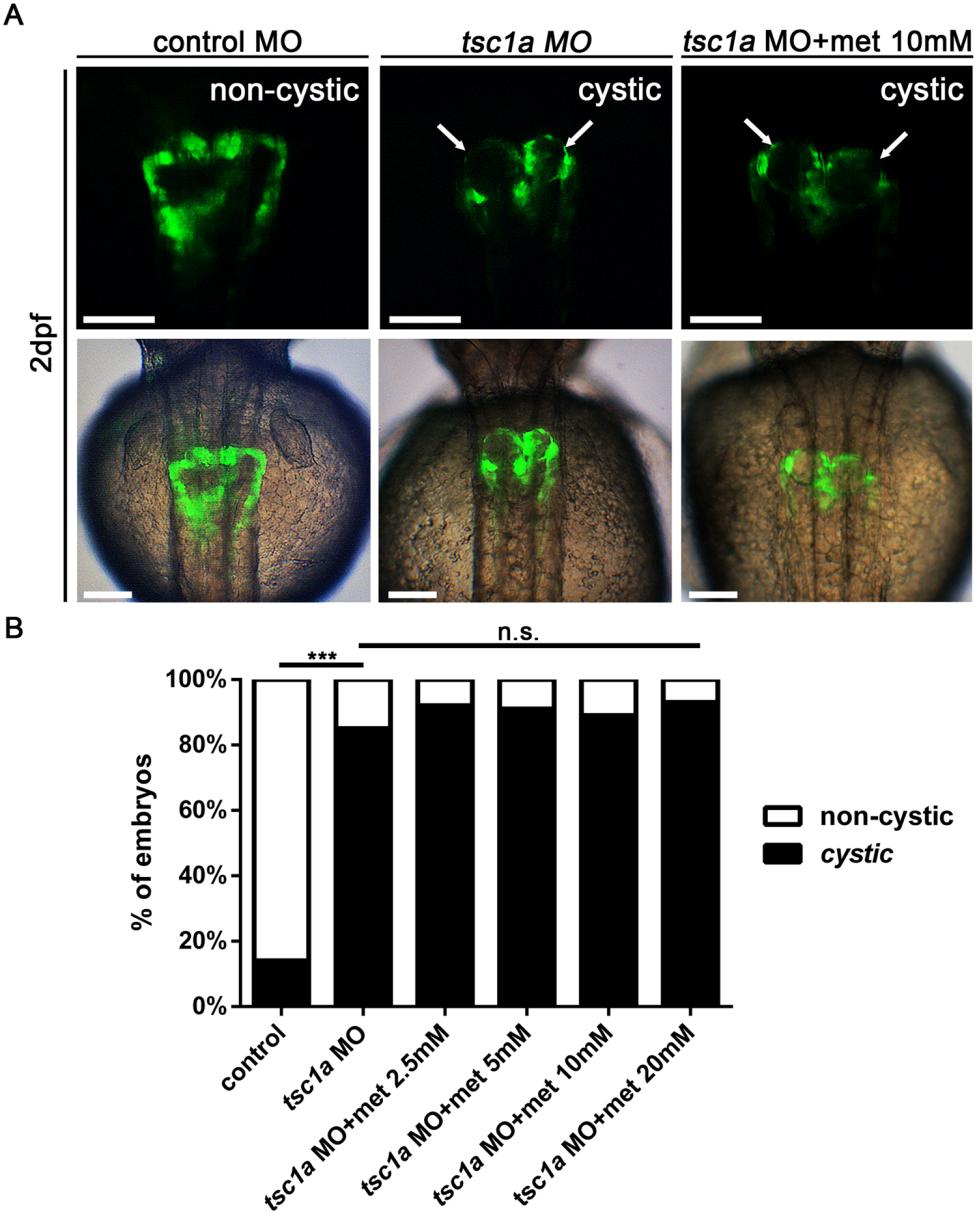
Metformin cannot rescue the cystic phenotype in *tsc1a* morphants. *Tg*(*wt1b:GFP*) *tsc1a* morphants were immersed in embryo media (E3) containing 2.5 to 20 mM metformin until 48 hpf. (**A**) Representative images indicating prominent cystic pronephros (arrows) in *Tg*(*wt1b:GFP*) *tsc1a* morphants. Metformin does not affect the formation and severity of pronephric cysts. The upper panels show fluorescence images (dorsal view, anterior to the top). The lower panels show overlays of fluorescence (green) and transmission (grey) images of the upper panels. Scale bar, 100 µm. (**B**) Comparison of the cystic phenotype in different treatment groups. Data represent two independent experiments (n = 28–46 per group). ***P < 0.001, n.s. = not significant.

A study indicated that the AMPK-dependent activation of tuberous sclerosis complex (TSC) 1 and 2 proteins plays a major role in the inhibition of the mTOR signalling pathway during energetic demands^39^. Therefore, we tested whether metformin prevented cyst initiation in the absence of TSC proteins. Morpholino knockdown of *tsc1a* induced severe glomerular cysts in zebrafish embryos at 48 hpf, as reported previously (Fig. 6A)^40^. Metformin apparently did not affect cyst formation in the *tsc1a* morphants (Fig. 6B). This observation is consistent with an AMPK/TSC-dependent mechanism underlying the prevention of cyst formation by metformin.

### Metformin enhances autophagy in the pronephros

**Figure 7:**
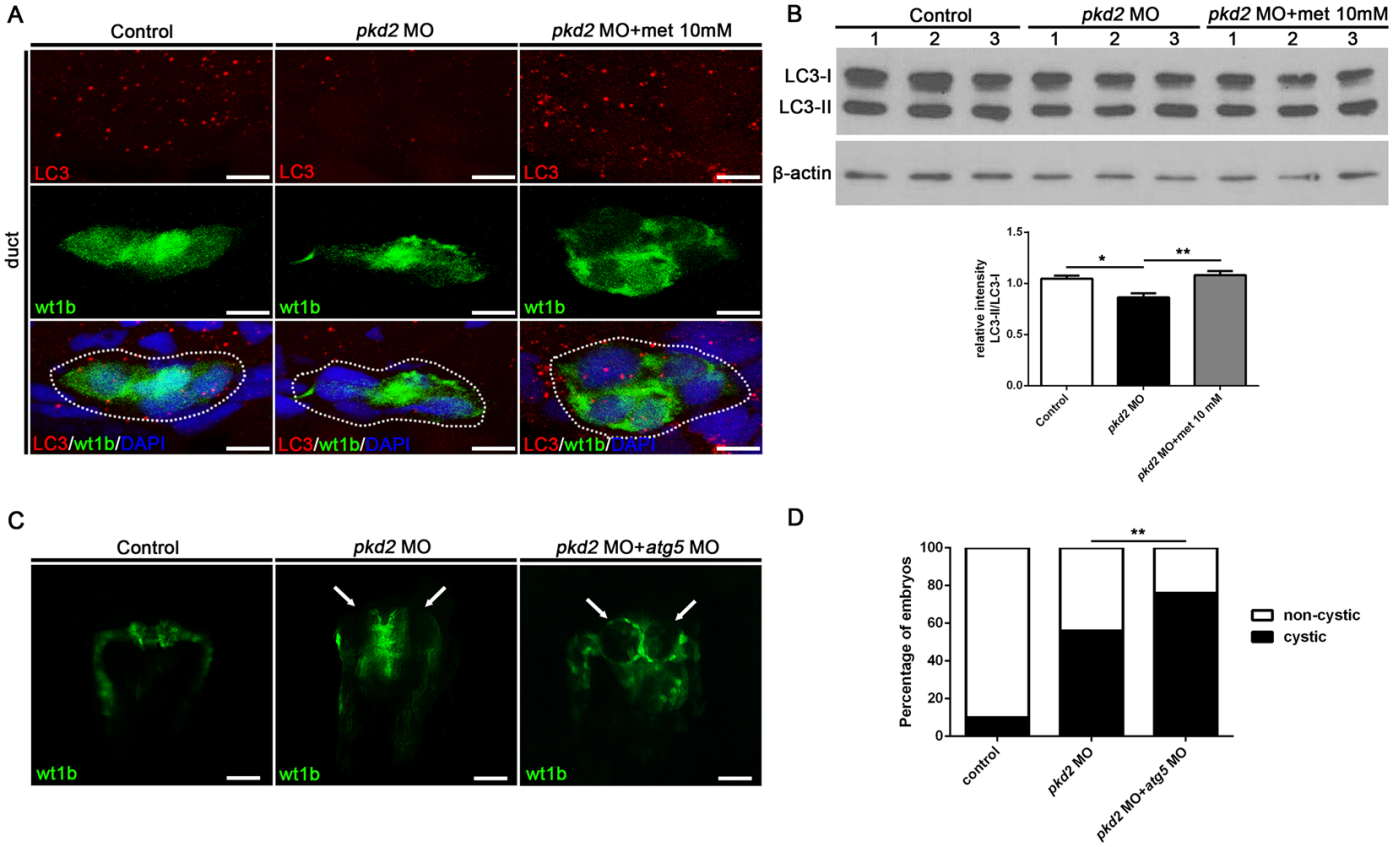
Metformin enhances autophagy in *Tg*(*wt1b:GFP*) *pkd2* morphants. (**A**) Representative confocal images of pronephric ducts (green, *wt1b:GFP*) in control MO-injected embryos, *pkd2* morphants, and *pkd2* morphants treated with metformin (10 mM) at 48 hpf (n = 4 per group). Transverse cryosections were stained for LC3 (red), and the nuclei were counterstained with DAPI (blue). Note the reduced expression of LC3 (orange dots, red and green merged) in the *pkd2* morphant and the rescue effect of metformin treatment. The dotted circle indicates the pronephric duct. Scale bar, 10 µm. (**B**) Quantification of the expression of LC3-II relative to LC3-I through Western blot analysis of total embryo lysates. ß-actin was used as a loading control; n = 8 from four independent experiments. *P < 0.05 compared with control; **P < 0.01 compared with untreated *pkd2* morphants. The uncropped blots are shown in Supplementary Fig. S2. (**C**) A combined knockdown of *atg5* and *pkd2* increased the frequency of cystic phenotype in the *Tg*(*wt1b:GFP*) *pkd2* morphants. Representative images of the pronephros in the control embryos, *pkd2* morphants, and *atg5*/*pkd2* double morphants are shown. Arrows indicate the pronephric cysts. (**D**) Comparison of the cystic phenotype in different groups. Data represent four independent experiments (n = 81, 86, and 87 per group). **P < 0.01.

Defective autophagy, an AMPK downstream cellular process that degrades cytoplasmic components for energy production, has been associated with the pathogenesis of ADPKD in recent studies^41^. Therefore, we examined whether metformin increased the autophagy activities in *pkd2* morphants. Autophagy activity was assessed by detecting the intracellular levels of microtubule-associated protein light chain 3 (LC3) through immunofluorescence and Western blot analysis^42^. As illustrated in Fig. 7A, the *pkd2* morphants exhibited lower LC3 staining in the pronephric ducts than did the control embryos, and the deficiency was rectified by metformin treatment. Consistent with these findings, the Western blot analysis indicated that the conversion of cytoplasmic LC3-I into LC3-II was significantly lower in the *pkd2* morphants than in the control embryos, and metformin treatment restored the autophagy activities (Fig. 7B and Supplementary Fig. S2). Furthermore, we observed that simultaneous knockdown of the autophagy gene *atg5* significantly increased the cystic phenotype in the *pkd2* morphants (Fig. 7C and D). Although the magnitudes of these differences were moderate and further studies are needed to confirm our findings, these results suggest that the activation of AMPK and restoration of autophagy constitute a plausible mechanism for the inhibition of cyst growth by metformin^43^.

## Discussion

The zebrafish has become an increasingly recognised model for identifying drug candidates for ADPKD treatment^44–47^. Chemical compound screening in zebrafish *pkd2* models indicated that histone deacetylase inhibitors inhibited cyst growth^48^. Candidate drugs that are effective in other PKD animal models, including rapamycin, roscovitine, and pasireotide, have been successfully validated using the zebrafish system^34, 49^. In the current study, we demonstrated that metformin activated the phosphorylation of AMPK and prevented cyst formation in a zebrafish *pkd2* model. In particular, we demonstrated that metformin inhibited epithelial cell proliferation and restored normal autophagy activity in the pronephric kidney. Furthermore, we demonstrated that metformin did not prevent cyst formation in *tsc1a* morphants resulting from knocking down the AMPK downstream target TSC1.

Metformin was shown to suppress cyst growth and fluid secretion through the inhibition of mTOR and CFTR in a previous study using MDCK cell cysts, mouse embryonic kidney explant cultures, and *Pkd1* mouse models^23^. In accordance with this study, we found that metformin inhibited early cystogenesis in zebrafish *pkd2* morphant embryos, indicating that metformin can inhibit cyst growth in both PKD1 and PKD2 animal models. These results support the hypothesis that metformin reduces cyst formation in the early stages of ADPKD. Interestingly, a previous study suggests that polycystin-2 deficient cell lines are less amenable to metformin and rapamycin than polycystin-1 deficient cell lines due to the less activation of mTOR pathway^24^. However, we observed a similar suppressive effect of metformin on the curvature phenotype in both *pkd1a*/*b* and *pkd2* morphants. The discrepancy could be explained by the differences between *in vivo* and *in vitro* cystic models, or metformin could have pleiotropic effects on multiple signalling pathways related to the PKD2 deficiency. Further studies will be required to clarify our findings.

One possible mechanism by which metformin prevents cyst formation is through the activation of AMPK and inhibition of cell proliferation. The AMPK signalling pathway plays a crucial role in maintaining normal kidney structure during nephron morphogenesis by affecting cell proliferation and migration^50, 51^. Activated AMPK restores the cell energy balance by shutting down the ATP-consuming synthesis pathways, and thus causes cells to switch from an anabolic to a catabolic state^18^. Metformin reduced cell proliferation in an AMPK-dependent manner in MDCK cysts grown in 3D collagen gels^23^. Similarly, intraperitoneal injection of metformin resulted in a reduction in the number of Ki67-positive epithelial cells in the cystic kidneys of *Pkd1* ^*flox*/*−*^ *Ksp-Cre* mice at postnatal day 7^23^. Furthermore, forced activation of AMPK by 2-deoxyglucose (2DG) restored normal extracellular signal-regulated kinase (ERK) activity, inhibited glycolysis, and reduced the cystic index and proliferation rate in *Pkd1* conditional knockout mice^9^. Our findings are consistent with these results and suggest that metformin may inhibit cystogenesis through an AMPK-dependent pathway.

Growing evidence substantiates the role of macrophages in promoting cyst growth in ADPKD. Macrophages were demonstrated to stimulate cell proliferation and cyst expansion in coculture experiments within a collagen matrix^52^. The recruitment and retention of renal macrophages contributes to the increased proliferation during cyst growth in ADPKD^10^. A previous study showed that metformin treatment reduced inflammatory cytokine production in peripheral blood mononuclear cells obtained from healthy volunteers^53^. Metformin also suppressed inflammation through activation of AMPK and phosphatase and tensin homolog (PTEN) in vascular smooth muscle cells^54^. In accordance with these findings, we observed that metformin reduced the recruitment of macrophages in *pkd2* morphants, which could be an alternative mechanism through which metformin reduces cell proliferation and cyst formation.

Another significant finding in this study is that metformin corrects the defective autophagy observed in *pkd2* morphants. Autophagy is a highly regulated cellular process that degrades and recycles intracellular proteins and organelles in lysosomes during metabolic stress or nutritional deprivation^55^. Our finding that *pkd2* morphants had defective autophagy is consistent with a previous study showing that *Pkd1* ^−/−^ mouse embryonic fibroblasts had insufficient autophagy activity upon glucose deprivation^9^. Furthermore, our results suggested that metformin might reduce cyst formation through the enhancement of autophagy in ADPKD^56^. Autophagy suppression could reduce the senescence of cyst-lining cells and lead to increased proliferation, apoptosis, and cyst growth^57^. Autophagy has also been shown to regulate the formation and function of primary cilia^58^. The detailed mechanisms that link autophagy and cyst growth require further study^59^.

The similarities in the cystic phenotype of *pkd2* and *tsc1a* morphants prompted us to investigate whether metformin could also inhibit cyst formation in this previously described model of tuberous sclerosis^40^. However, our results demonstrated that metformin could not rescue the cystic phenotype of the *tsc1a* morphants. This observation is consistent with an AMPK-dependent effect of metformin because TSC1 is a known downstream target of AMPK. Long-term treatment with metformin also failed to suppress renal tumours in a *Tsc1* ^+/*−*^ mouse model^60^. We were unable to further determine the relative contribution of AMPK activation in the inhibition of cystogenesis because of a lack of a proper morpholino or specific antagonist to reduce AMPK activity without interfering with the development of zebrafish embryos^38^. Therefore, we cannot exclude the possibility that metformin inhibits cystogenesis through an AMPK-independent effect^18, 61^.

Our data contribute to a growing body of evidence that metabolic abnormalities are crucial in the pathogenesis of ADPKD^50, 62^. Inhibition of aerobic glycolysis by using 2DG suppressed cell proliferation, leukocyte infiltration, and cyst formation in mouse models of PKD1^9^. Mild to moderate food restriction was demonstrated to activate AMPK and reduce cyst area, renal fibrosis, and inflammation^63^. These studies support a new therapeutic strategy for ADPKD, which involves reprogramming of cellular metabolism^64^.

Although our results support the hypothesis that metformin is a potential treatment for ADPKD, metformin could cause lactic acidosis in patients with advanced renal failure^18^. Therefore, the inhibitory effect of metformin on cyst growth in animal models requires confirmation in future clinical studies. A randomised, double-blind clinical trial evaluating metformin in the treatment of patients with ADPKD was started in 2016 and is expected to be completed in the next few years (ClinicalTrials.gov Identifier: NCT02656017).

In conclusion, metformin reduces cyst formation in *pkd2-*deficient zebrafish embryos. The data suggest that metformin may prevent cyst formation through activation of the AMPK pathway and modulation of defective cellular events such as proliferation and autophagy. The findings also indicate the therapeutic potential of metformin in treatment of ADPKD at the early stages. Further studies are required to assess whether metformin can improve clinical outcomes in patients with ADPKD.

## Methods

### Zebrafish maintenance

Animal experiments were approved by the Chang Gung University Institutional Animal Care and Use Committee. The investigation conformed to the National Institutes of Health Guide for the Care and Use of Laboratory Animals. Zebrafish and embryos were maintained according to standard procedures^65^. The embryos were staged according to hours post fertilisation. We used the *Tg*(*wt1b:GFP*) line (kindly provided by Prof. Christoph Englert, Fritz Lipmann Institute, Jena, Germany) for *in vivo* observation of pronephric cysts^25–27, 66^.

### Morpholino injection

Zebrafish embryos at the one- or two-cell stage were microinjected with 0.125 mM antisense MO. MOs were obtained from Gene Tools (Philomath, OR) and had the following sequences: *pkd2* ATG-MO (5′-AGGACGAACGCGACTGGAGCTCATC-3′)^29^, *pkd2* 5-mismatch MO (5′-AGCACCAACCCGACTGCACCTCATC-3′), *tsc1a* ATG-MO (5′-CCATAGTTGTGCAGGACAGTGGGCA-3′)^40^, *tsc1a* 5-mismatch MO (5′-CCATACTTCTGCAGCACACTGGCCA-3′), *pkd1a* exon 8 splice-MO MO: (5′-GATCTGAGGACTCACTGTGTGATTT-3′)^30^, *pkd1b* exon 45 splice-MO MO: (5′-ACATGATATTTGTACCTCTTTGGTT-3′)^30^, and *atg5* ATG-MO (5′-CACATCCTTGTCATCTGCCATTATC-3′)^67^.

### Drug treatment for zebrafish

*Pkd2* morphant embryos (n = 20–30) were incubated in 6-cm Petri dishes with 8 mL of the E3 medium (5 mM NaCl, 0.17 mM KCl, 0.4 mM CaCl_2_, and 0.16 mM MgSO_4_) in an incubator at 28.5 °C. Metformin (Sigma) was added to the E3 medium at final concentrations of 5 to 20 mM at 4 hpf, a time point before the development of body curvature and pronephric cysts according to previous studies^30, 48^. At 48 hpf, the living embryos were anaesthetised with tricaine (0.2 mg/mL) and oriented in 3% methylcellulose (Sigma). The presence of pronephric cysts was determined using fluorescence microscopy^25–27^. The dorsal body curvature phenotype of *pkd2* morphants was categorised as normal, mild (tail angle less than 90°), or severe (tail curvature angle greater than 90°)^48^.

### Immunofluorescence

Embryos were fixed overnight in 4% paraformaldehyde at 4 °C. Immunostaining was performed in whole-mount embryos as described previously^68^. A rabbit anti-PH3 antibody (1:200, Millipore) was used to label proliferating cells, and a mouse anti-α6 F antibody (1:200, Developmental Studies Hybridoma Bank) was used to label pronephric epithelial cells. LC3, an autophagosome marker, was labelled using a rabbit anti-LC3B antibody (1:200, Novus Biologicals). Secondary antibodies used were the Alexa Fluor 594 goat antirabbit IgG and Alexa Fluor 488 goat antimouse IgG (1:500, Molecular Probes). Proliferating cells in the pronephric ducts were counted in anti-PH3 and anti-α6 F stained whole-mount embryos using fluorescence microscopy. For confocal imaging, embryos were flat-mounted and the alpha 6 F and PH3 signals were recorded in z-series stacks using a Zeiss LSM 510 confocal microscope. For analysis of LC3 staining, the immunostained embryos were embedded in the OTC medium and cryosectioned through the pronephros. The sections were mounted in Vectashield (Vector Laboratories) with DAPI and imaged using confocal microscopy.

### Histology

The embryos were fixed in 4% paraformaldehyde overnight at 4 °C and embedded in glycolmethacrylate (JB-4; Polyscience). Serial sections (4 μm) were cut and stained with Hematoxylin and Eosin (H & E).

### Whole-mount *in situ* hybridisation

The embryos were fixed overnight in 4% paraformaldehyde at 4 °C. Whole-mount *in situ* hybridisation was performed according to published protocols^65^. Antisense digoxigenin-labelled RNA probes were synthesised from linearised plasmid templates containing *l-plastin* and *mpx* cDNAs.

### Western blot analysis

Proteins were extracted from whole embryos for Western blot analysis using standard protocols^38, 69^. Primary antibodies, namely anti-phospho-AMPKα (Thr172) (1:1000, Cell Signalling), anti-AMPKα (1: 1000, Cell Signalling), anti-LC3B (1:10000, Novus Biologicals), and anti-β-actin (AC-15) (1: 10000, Abcam) antibodies, were used overnight at 4 °C. Horseradish peroxidase-conjugated secondary antibodies were used for 1 h at room temperature. The signals were detected through enhanced chemiluminescence.

### Statistical analysis

Values are expressed as the mean ± SEM. Comparisons between groups were performed using the Student’s *t* test or ANOVA followed by Dunnett’s multiple comparison test. Values that were not normally distributed were analysed using the Kruskal–Wallis test followed by Dunnett’s multiple comparison test. Categorical variables were analysed using Fisher’s exact test or the chi-square test. *P* values less than 0.05 were considered statistically significant. All analyses were performed using GraphPad Prism 5.0 (GraphPad, La Jolla, CA, USA).

## Electronic supplementary material

Supplementary Information
