# Supplementary material for: Metformin Inhibits Cyst Formation in a Zebrafish Model of Polycystin-2 Deficiency
Source: Sci Rep. 2017 Aug 2;7:7161. doi: 10.1038/s41598-017-07300-x (PMC5541071; doi:10.1038/s41598-017-07300-x)
Supplement: Supplementary file 1 — Supplementary Information [file 41598_2017_7300_MOESM1_ESM.doc]

**Supplementary information**

**Metformin Inhibits Cyst Formation in a Zebrafish Model of Polycystin-2 Deficiency**

Ming-Yang Chang, Tsu-Lin Ma, Cheng-Chieh Hung, Ya-Chung Tian, Yung-Chang Chen, Chih-Wei Yang. Yi-Chuan Cheng.

**Supplementary Figures**

**
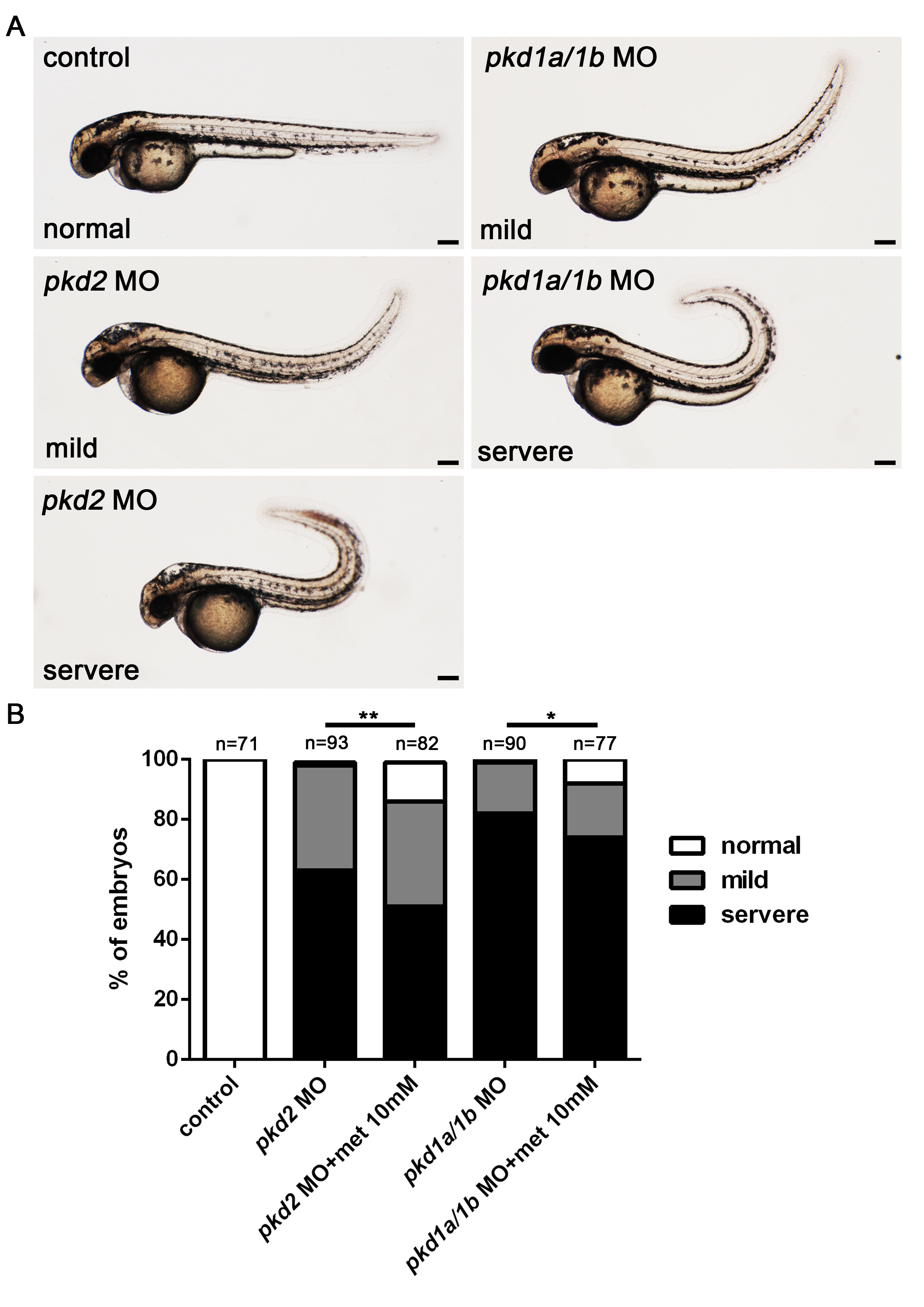
**

**Supplementary Fig. S1. Metformin ameliorates dorsal axis curvature in *pkd1a/b* and *pkd2* morphants.** Zebrafish embryos were treated with metformin (10 mM) in supplemented E3 media between 4 and 48 hours post fertilisation (hpf). (A) Representative images showing the common body axis of a control embryo, and the mild and severe dorsal curvature phenotypes of *pkd1a/b* and *pkd2* morphants at 48 hpf. (B) Comparative frequencies of the dorsal curvature phenotype in the *pkd1a/b* and *pkd2* morphants with and without metformin treatment. Data represent two independent experiments. **P* < 0.05, ***P* < 0.01. Scale bar, 200 µm.


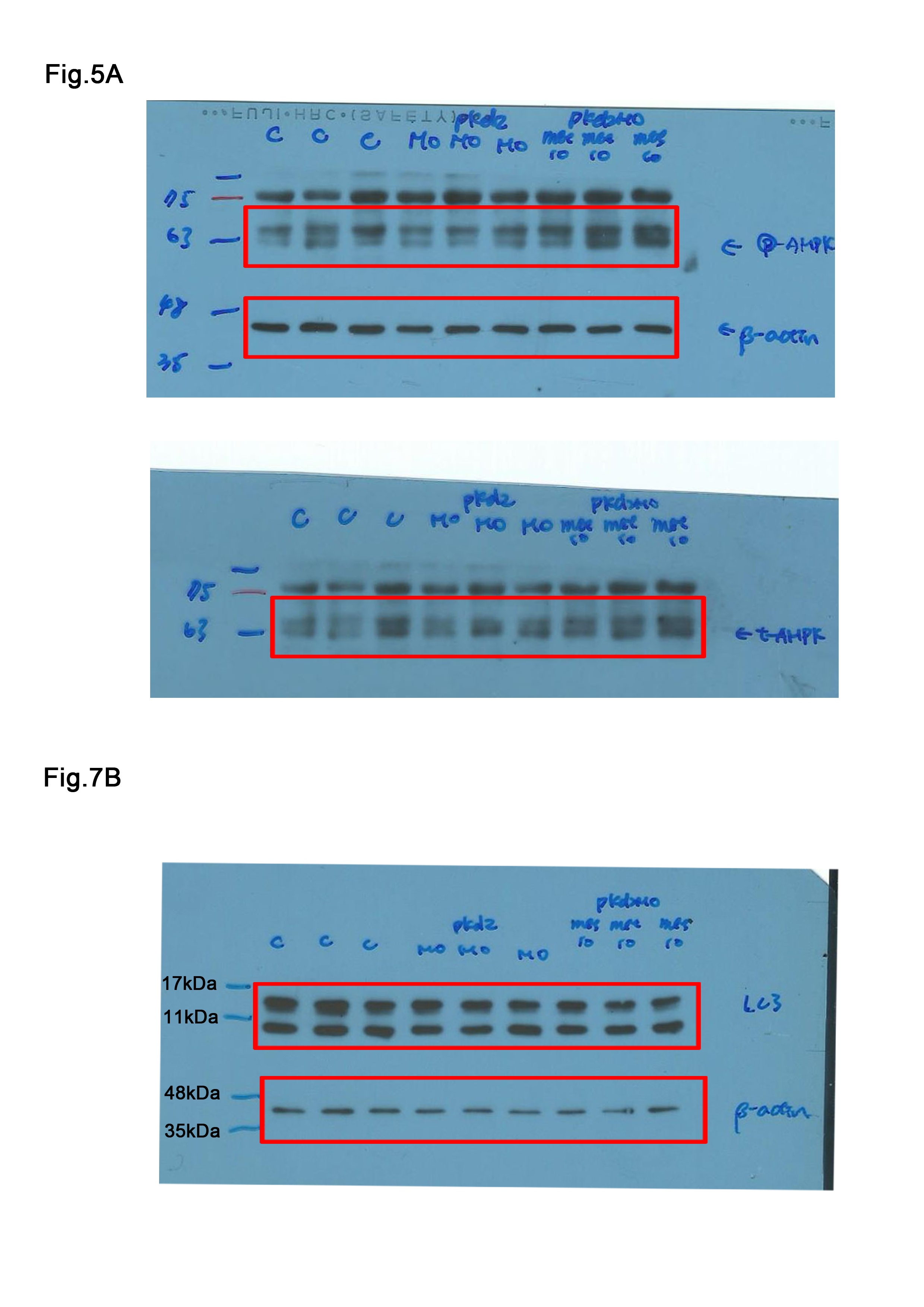


**Supplementary Fig. S2.** Uncropped blots corresponding to the cropped images reported in Fig.5A and Fig.7B.
